# Retraction Note: Counter-ion dependent, longitudinal unzipping of multi-walled carbon nanotubes to highly conductive and transparent graphene nanoribbons

**DOI:** 10.1038/s41598-025-31859-5

**Published:** 2025-12-15

**Authors:** Dhanraj B. Shinde, Mainak Majumder, Vijayamohanan K. Pillai

**Affiliations:** 1 Physical & Materials Chemistry Division, National Chemical Laboratory, Pune, 411 008 India; 2 Nanoscale Science and Engineering Laboratory (NSEL) and Mechanical and Aerospace Engineering Department, Monash University, Clayton, VIC 3800 Australia; 3 Central Electrochemical Research Institute (CSIR-CECRI), Karaikudi, 630 006 India

Retraction of: *Scientific Reports* 10.1038/srep04363, published online 13 March 2014

The Editors have retracted this Article.

After publication, concerns were raised regarding the veracity of the XRD patterns and Raman spectra reported in Figures 3a and 3b, respectively.

The Authors provided original data on request from the Editors. However, the Editors found that the XRD patterns and Raman spectra differ substantially from those presented in the paper. The Editors therefore no longer have confidence in the research presented in this work.

The Authors disagree with this retraction.

